# A Nationwide Survey of Italian University Students: Exploring the Influences of Sustainable Dietary Behaviors, Lifestyle, and Sociodemographic Factors on Adherence to the Mediterranean Diet

**DOI:** 10.3390/nu17121988

**Published:** 2025-06-12

**Authors:** Cinzia Franchini, Beatrice Biasini, Giovanni Sogari, Rungsaran Wongprawmas, Giulia Andreani, Miguel I. Gómez, Davide Menozzi, Cristina Mora, Francesca Scazzina, Alice Rosi

**Affiliations:** 1 Department of Food and Drug, University of Parma, 43124 Parma, Italy; cinzia.franchini@unipr.it (C.F.); beatrice.biasini@unipr.it (B.B.); giovanni.sogari@unipr.it (G.S.); rungsaran.wongprawmas@unipr.it (R.W.); giulia.andreani@unipr.it (G.A.); davide.menozzi@unipr.it (D.M.); cristina.mora@unipr.it (C.M.); alice.rosi@unipr.it (A.R.); 2 Charles H. Dyson School of Applied Economics and Management, Cornell University, Ithaca, NY 14853, USA; mig7@cornell.edu

**Keywords:** Mediterranean diet, SHED index, sustainable diets, healthy dietary patterns, plant-based consumption, young adults

## Abstract

Background/Objectives: Recent decades have seen a remarkable westernization of diets and a decline in adherence to the Mediterranean diet (MD). This study examined the eating habits of a representative sample of Italian university students to identify the determinants of adherence to the MD and the most relevant actions to improve their well-being. Methods: The Mediterranean Diet Quality Index for Children and Adolescents (KIDMED) and Sustainable Healthy Diet (SHED) index questionnaires were used to explore MD adherence as the primary outcome, and dietary behavior sustainability, respectively. Sociodemographic, anthropometric, and lifestyle information was also collected. Results: The final sample included 1434 subjects (18–24; 60% female). The median (IQR) KIDMED score was 6.0 (4.0–8.0) and 33% showed a high adherence to the MD. Having breakfast, eating fruit and vegetables at least once a day, consuming pasta or other grains almost daily, and using olive oil at home were among the most common positive aspects of students’ diets. However, regular consumption of fish, nuts, legumes, dairy products, and a second serving of fruit and vegetables was less prevalent. Having an active lifestyle, eating more plant products, and having more sustainable dietary behaviors in terms of the SHED index, were the main determinants of a high KIDMED score. Likewise, graduate students, daily consumers of plant-based meat alternatives, and students attending university canteens daily were more likely to adopt healthier diets. Conclusions: Future actions are crucial to create a food environment that facilitates healthy and sustainable dietary choices among young adults, such as improving the nutritional quality of processed products and regulating their promotion, as well as implementing initiatives to improve university food services and encourage the use of campus dining facilities.

## 1. Introduction

As suggested by the Lancet Commission, obesity, malnutrition, and climate change should not be considered individually, but as a “Global Syndemic” and tackled with a holistic approach to achieving global targets of human and ecosystem health and well-being, economic flourishing, and social equity. Along with transportation, urban design, and land use, food and agriculture are leading drivers of this damaging synergy [1], and adopting healthy and sustainable diets represents a great opportunity to reduce the environmental impacts of food systems and improve health outcomes [2]. To give a concrete example, extensive evidence supports [3,4,5,6,7,8,9,10] the Mediterranean diet (MD) as a well-established model of sustainable nutrition that enhances human and planet health. This traditional dietary pattern unites the various regions of the Mediterranean basin and is characterized by a moderate intake of animal products in favor of an abundant consumption of plant foods such as fruit, vegetables, cereals, legumes, nuts, and seeds. Another peculiarity of the MD is the use of olive oil as the principal seasoning [7]. The MD principles are in line with the Planetary Health Diet (PHD) recently defined by the EAT-Lancet Commission. This panel of leading experts delineated a combination of intake ranges for different types of food to achieve sustainable food systems and human well-being [2]. Following the establishment of these recommendations, several studies have developed new dietary quality scores [11,12,13,14,15], such as the one defined by the Sustainable Healthy Diet (SHED) index settled by Tepper and colleagues in 2021 using both PHD guidelines and MD principles [16].

The need to promote sustainable nutrition and establish indices to measure its adoption in populations has been heightened by dietary and lifestyle changes occurring across the globe [15]. In particular, the last decades experienced remarkable increases in the quantity and availability of foods in developed countries, resulting in higher access to animal-based and energy-dense foods. This phenomenon has contributed to the globalization process of diets, shifting people’s eating habits toward Western-type dietary patterns across the world [17,18]. Since the 2000s, the process of diet westernization has been observed also in Mediterranean regions, including Italy [19]. More recent evidence confirms this progressive shift away from the MD in Italian adults [20,21,22,23,24]. In parallel to food globalization, other factors, such as the economic crisis, and social and cultural influences have greatly affected people’s way of life [19,25] and contributed to a declining adherence to the MD in all age groups, including young generations [26]. At the same time, a recent study [27] pointed out that young adults aged 18 to 24 years are the population group with the highest risk of becoming overweight or obese in the next ten years of life, regardless of gender, ethnicity, and geographic or socioeconomic area, and Italy is no exception to this trend [28]. These data suggest the urgent need for actions targeting this age group to prevent obesity and its long-term health implications [27].

Given the growing number of young adults enrolled in university programs worldwide [29], implementing interventions through the academic system makes it possible to reach a large number of individuals. In addition, the existing evidence identifies college life as a time of increased independence and stress related to the study load, social expectations, economic restrictions, and inaccessibility of quality food [30,31]. All these factors, along with unreliable dietary information [32] and advertising of unhealthy foods and beverages provided by the mass media [33], expose university students to numerous health risks [30,31], including an increased likelihood of establishing unhealthy eating habits [34,35] and developing excess weight conditions and related diseases [36,37].

In this context, the present study aimed to describe the current dietary habits of a nationally representative sample of Italian university students. Specifically, it sought to assess the influences of sustainable dietary behaviors, lifestyle, and sociodemographic characteristics on adherence to the MD. The findings are intended to guide the design of targeted initiatives to promote the well-being of university communities.

## 2. Materials and Methods

### 2.1. Participants and Study Design

A representative sample of Italian young adults between 18 and 24 years old registered in a university program were enlisted via a marketing agency (Dynata^TM^: Shelton, CT, USA) in May 2022. To achieve a nationally representative sample of the Italian university population, at least 1400 participants had to participate in the study. This criterion was established based on the number of university students (*n* = 1,627,780) reported in the data record provided by the Italian National Institute of Statistics (ISTAT) [38]. The sample size calculation was performed using the G*Power 3.1.9.7 calculator [39], and both gender and regional distributions were considered to obtain a representative sample of the Italian university population. Participants were asked to fill out a self-administered questionnaire using the Qualtrics online platform (Qualtrics software, version [May 2022] of Qualtrics, Copyright © [2022] Qualtrics). The time needed to complete the survey was considered a quality criterion, and respondents who took less than 40% of the median time or more than 1 h to fill out the questionnaire were excluded [40,41,42]. This study was approved by the local institutional review board in Italy (Research Ethics Board, University of Parma, REB 85797) and conducted according to the ethical principles stated in the Declaration of Helsinki.

### 2.2. Data Collection

The data collection methods and tools have been previously described in detail [43]. Briefly, the KIDMED [44] and SHED index [16] questionnaires were administered to explore the adherence to the MD and the sustainability of students’ dietary behaviors, respectively. The KIDMED consists of 16 yes/no questions developed on the dietary principles of the MD. Based on the cut-offs defined by the authors [44], respondents were classified into three adherence levels: low (total score  ≤  3 points), medium (total score of 4–7 points), or high (total score  ≥  8 points). The SHED index includes six sub-scores rated using three different Likert scales: the Healthy Eating (HE) and Sustainable Eating (SE) sections were scored on a 4-point Likert scale, ranging from “Almost never true” (0 points) to “Almost always true” (3 points); the Fruits and Vegetable Purchasing Location (BFV) and Water sections were scored on a 4-point Likert scale, ranging from “Never” (0 points) to “Most of the time” (3 points); and Ready Meals and Sodas were scored on 6-point Likert scale from “Never” (0 points) to “Daily or almost daily” (5 points). A specific data processing procedure provided by the authors was followed to calculate the total score [16]. The total score was obtained by summing each sub-score, and higher scores indicated more sustainable eating behaviors. Sociodemographic information was provided by each subject along with self-reported body weight and height. Anthropometric data were considered as continuous variables and used to calculate students’ Body Mass Index (BMI), as weight in kilograms divided by the square of height in meters (kg/m^2^), and to estimate their weight status according to standard cutoffs of the World Health Organization (WHO): underweight (BMI < 18.5 kg/m^2^), normal weight (18.5–24.9 kg/m^2^), overweight (25.0–29.9 kg/m^2^), and obesity (BMI ≥ 30.0 kg/m^2^) [45]. In addition, adherence to WHO physical activity guidelines (World Health Organization, 2024) [46] was assessed through the closed-ended version of the Nordic Physical Activity Questionnaire (NPAQ-short) [47]. Only the participants who reported performing a minimum of 150–300 min of Moderate to Vigorous Physical Activity (MVPA), 60–90 min of Vigorous Physical Activity (VPA), or a combination of 90–150 min of MVPA and 30–60 min of VPA in a typical week were classified as compliant. Lastly, more details were gathered on dietary habits, such as the frequency of attending university cafeterias over the 6 months prior to the study, participants’ willingness to select healthy and sustainable dishes, the dietary pattern adopted (e.g., omnivorous, flexitarian, etc.), and their frequency of consumption of alternative ultra-processed meat foods. The latter question was adapted from Ohlau and colleagues [48].

### 2.3. Data Reporting and Statistical Analysis

All statistical analyses were performed with the IBM SPSS Statistics, version 29.0 (Armonk, NY, USA: IBM Corp.), and a *p*-value less than 0.05 was considered statistically significant. Based on the Kolmogorov–Smirnov test, the normality of the data distribution was rejected, and continuous variables were reported as medians and interquartile ranges (IQRs), while categorical variables were expressed as absolute numbers and percentages. Cronbach’s alpha reliability test and Spearman’s correlation test between the single sub-scores and the total score were used to test the internal consistency and sub-scores’ correlations with SHED index questionnaires, respectively. Given the non-normal distribution of the data, the non-parametric Kruskal–Wallis H test with pairwise comparisons was applied to investigate differences among genders (men vs. women vs. non-binary gender) and subjects with different levels of adherence to the MD (low vs. medium vs. high). In addition, Pearson’s Chi-square test (χ^2^) was used to explore associations among categorical variables. Based on these results, the variables for which significant associations were found were used as independent factors in univariate and multivariate logistic regression analyses. These statistics were performed to identify the factors that most increased the probability of having a high adherence to the MD (dependent variable). In addition, the STROBE-nut reporting guidelines checklist [49] was used to improve the clarity of data reporting (Supplementary Table S1—Online Resource 1).

## 3. Results

### 3.1. Characteristics of the Sample and Differences Among Genders

A total of 1488 subjects correctly completed the online survey. Low-quality records, i.e., too-fast (*n* = 17) and too-slow (*n* = 26 respondents), were removed due to the potential unreliability of responses, suggesting a lack of seriousness in completing the questionnaire [42,50]. In addition, 11 participants report an unrealistic height or body weight and were therefore removed from the analysis. The final sample included 1434 young adults, representative of university students residing in Italy. Participants’ data are shown in Table 1 for the total sample and by gender.

The age range of the sample was 18–24 years and women were the majority. Most students reported anthropometric measurements within the normal BMI range, while 21% were found to be overweight or obese. More than half of the students came from southern regions or islands, but considering the geographical location of the university, the distribution of the sample was more equally distributed across the country. Most of the students were undergraduates, almost half were enrolled in human–social university programs, and more than one-fourth followed a course in the scientific–technological field, whereas food science and medicine programs were attended by less than 20% of students. In addition, about 35% did not live with their parents at the time of the survey, 18% reported not having enough to get by or preferred not to answer the question on their economic status, and the attendance of the university dining services over the 6 months prior to the study was null or very low in more than half of the sample (46%). In addition, MVPA recommendations were met by about half of the sample. Overall, men had a greater tendency to be overweight than women and the non-binary gender, who tended to be more likely underweight (*p* < 0.001). However, men showed higher compliance with physical activity recommendations (*p* < 0.001). With regards to dietary habits, the median KIDMED score was 6.0 (4.0–8.0) and the group of respondents having a medium level of adherence was the most prevalent (55%).

Delving into the KIDMED questionnaire items for the total sample, 76% of the students consumed one fruit or fresh fruit juice in their daily diet, and 30% of them ate a second fruit every day. Vegetables were consumed once a day by 74% of the participants, and 48% reported consuming a second serving every day. In addition, 51% ate fish at least 2–3 times a week, and 65% consumed legumes more than once a week. Cereals such as pasta or rice were consumed by 74% of students almost every day. A total of 82% of students had breakfast every day, of which 60% ate cereals or grains, 64% dairy products, and 54% baked goods or pastries for breakfast. Also, 23% stated consuming two yogurts and/or some cheese every day. In addition, 47% of participants regularly consumed nuts, and 92% used olive oil at home. Only 13% of students reported going to fast food restaurants more than once a week, and 23% ate sweets and candies several times a day. No significant differences were found between genders in terms of the KIDMED score and level of MD adherence. However, men were found to be more used to eating a second fruit every day, consuming pasta or rice almost every day and nuts regularly, as well as eating two yogurts and or some cheese daily. In contrast, women and nonbinary respondents consumed more vegetables, used olive oil at home more often, and went to fast-food restaurants less frequently (Supplementary Figure S1—Online Resource 2). When considering regional differences across Italian macro-areas, students from northern regions were more likely to consume more than one serving of fruits and vegetables per day, to eat nuts regularly, and to eat two yogurts and/or some cheese daily. Conversely, students living in the central and southern regions, as well as the islands, reported a more frequent intake or pasta and grains, along with a higher use of olive oil. Finally, students from southern regions and islands were more used to consume pulses more than once a week (Supplementary Figure S2—Online Resource 3).

As for the SHED index score and sub-scores, the median values shown in Table 1 highlighted more sustainable behaviors among women. More details about students’ dietary behaviors are reported by gender in Figure S3 (Supplementary Figure S3—Online Resource 4). To summarize the significant differences, a higher share of men preferred animal-based foods over plant-based ones and were more used to eating away from home and drinking tap water; a higher percentage of women declared eating five servings of fruit and vegetables per day, preferring crops with little or no pesticide use, and consuming self-cooked meals; as for non-binary consumers, they were more likely to prefer plant-based foods and avoid meat, to separate waste, to consume organic produce, to buy fruit and vegetables directly from the farm or market, to drink mostly water, and to limit sweet and soft drinks, even if most of them stated that they drank diet beverages often or daily. As for reliability of the questionnaire, our preliminary analysis showed a good level of correlation between single sub-scores and the total score (Spearman’s rank correlation coefficient > 0.3, *p* < 0.001 for all), except for the Soda score. In addition, overall internal consistency indicated a good reliability of the tool (Cronbach’s alpha = 0.71). However, certain constructs (i.e., BVF, Ready Meals, Water, and Soda) showed lower Cronbach’s alpha values, reflecting poor reliability (Supplementary Table S2—Online Resource 5).

Moreover, the students’ diet was on average 50% plant-based. However, only the minority declared being flexitarian, pescatarian, vegetarian, or vegan, and this percentage was significantly greater in women and non-binary respondents, who were also more willing to purchase and consume healthy and sustainable dishes (*p* < 0.001). Finally, 55% of the total sample consumed plant-based ultra-processed meat alternative foods and, of these, 13% consumed them weekly.

### 3.2. Differences Among Subjects with Different Levels of Adherence to the Mediterranean Diet

Comparisons among groups with different levels of adherence to the MD are presented in Table 2. Summarizing the significant differences, participants with low adherence were younger (*p* < 0.001) and less educated (*p* = 0.002). Also, adherence to the MD was associated with the financial situation (*p* = 0.022), weight status (*p* = 0.031), and compliance with MVPA guidelines (*p <* 0.001). As for food-related habits, significant associations were highlighted for the type of dietary pattern (*p <* 0.001), willingness to purchase and consume healthy and sustainable dishes (*p* < 0.001), and the habit of eating plant-based ultra-processed meat alternatives foods (*p* = 0.003). These results are in line with the self-reported percentage of plant-based foods in the diet, which was significantly greater in participants with higher MD scores (*p* < 0.001).

**Figure 1:**
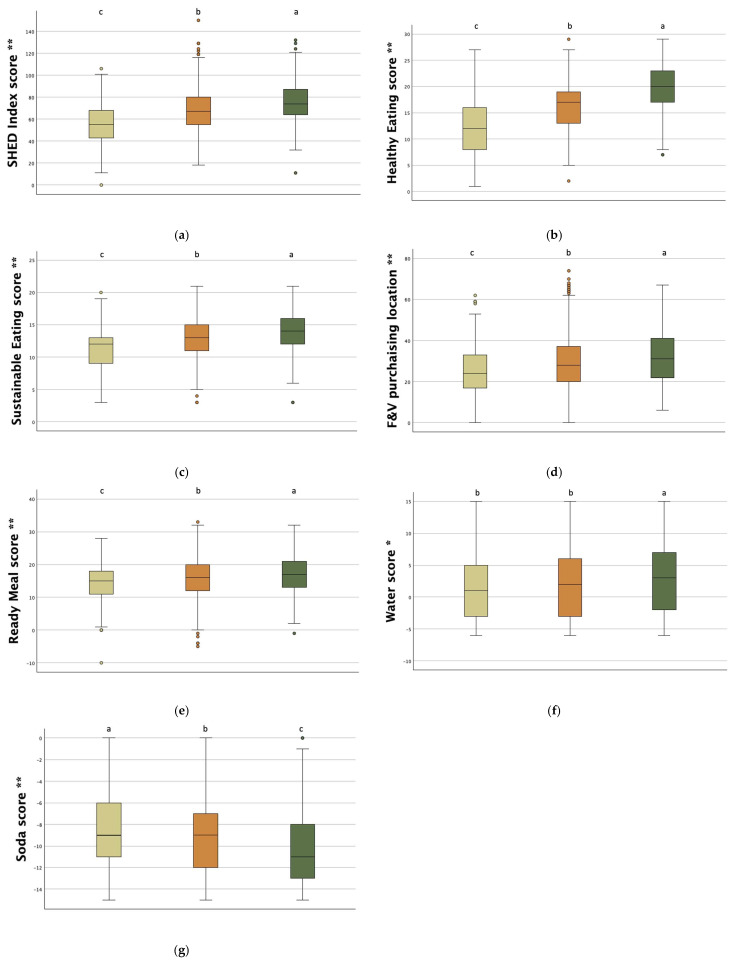
Sustainability of dietary behaviors. (**a**) SHED index score; (**b**) Healthy Eating score; (**c**) Sustainable Eating score; (**d**) F&V purchasing location; (**e**) Ready Meal score; (**f**) Water score; (**g**) Soda score. The total score and sub-scores are reported by the level of adherence to the MD (low−tan; medium−orange; high−dark green). Main effect from Kruskal—Wallis test with the Bonferroni post hoc test: * *p* < 0.01 and ** *p* < 0.001. Different letters indicate significantly different values.

In Figure 1, the SHED score and sub-scores are reported by the level of adherence to the MD. By comparing the three groups (low, medium, and high), the SHED index score (Figure 1a) and sub-scores (Figure 1b–f) were significantly higher in participants with higher adherence to the MD (all *p* < 0.001, but the Water score *p* = 0.004), except for the Soda score (Figure 1g), for which a significant but inverse association was found. Delving into the responses to the single items of the SHED index questionnaire provided in Figure S3 (Supplementary Figure S3—Online Resource 4), it can be noted that the Soda score is mainly driven by the item on the consumption of artificially sweetened beverages, with about 40% of respondents declaring that they consume them on a daily basis, regardless of gender.

### 3.3. Predictors of Adherence to the Mediterranean Diet

In Table 3, logistic regression analyses revealed the main determinants of adherence to the MD. Our findings corroborated the strong correlation between the weight status and the adoption of healthy habits. Thus, students with obesity and having a lower physical activity level were less likely to have high KIDMED scores. On the contrary, having a higher SHED index score, with their diet relying mainly on plant-based foods by following vegetarian eating patterns and being prone to purchasing and eating healthy and sustainable dishes, were found to be the main features of the students with high adherence to the MD. From the univariate analysis, other factors positively influenced the probability of being in the high adherence to the MD group, including being a graduate student, daily consumption of plant-based ultra-processed meat alternatives foods, and attending the university canteen on a day-to-day basis.

## 4. Discussion

This cross-sectional study fills a literature gap by providing insights into the eating habits of Italian university students not previously investigated in a nationally representative population. The novelty of the present research is the simultaneous application of the KIDMED and the SHED index scores, intending to provide a more comprehensive perspective on the healthfulness and sustainability of young adults’ dietary behaviors in Italy. As might be expected, Italian students showed greater adherence to the MD than the US students enrolled in the same study (high adherence 33% and 20%, in Italy and the US, respectively) [43]. Considering the Mediterranean regions, a recent literature review reported a low adherence and a progressive shift away from the MD principles among different populations of university students [26]. Focusing on the local context, our findings showed a more prevalent medium-to-high adherence to the MD than previous studies conducted on student populations in central [51] and southern Italy [52], whereas, for the northern area, the results in the literature are mixed [52,53,54]. It is worth noting that those samples [51,52,53,54] were not nationally representative and measured the adherence to the MD by applying scores other than the KIDMED.

Consistent with what has been reported by Lo Moro and colleagues for northern Italy [53], better adherence to the MD was associated with an extensive consumption of plant-based foods and the adoption of a vegetarian or vegan dietary pattern as well as a proper level of physical activity, whereas a lower adherence was related to obesity. Age was poorly correlated with adherence to the MD but, in our case, it might be due to the relatively small age range of our sample. The positive association between healthy eating habits and active lifestyle has already been reported in the literature in different populations of university students [43,52,55]. As for the weight status, the percentage of overweight participants is similar to that nationwide observed among young adults aged 18–24 (18%) in 2019, and lower than the European average (25%) [28]. Contrary to previous studies [51,53], this survey did not reveal any significant differences between genders in terms of adherence to the MD; however, considering the SHED index score and other food-related habits, men appeared to have less sustainable dietary behaviors and to consume fewer healthy and sustainable dishes. These findings are coherent with the existing literature showing that women are more willing to adopt more climate-sustainable diets, both considering the general population [56,57,58] and Italian university students [59]. However, regardless of gender, a significant association was identified between the SHED index score and the level of adherence to the MD. Such a result is consistent with four previous studies conducted in Mediterranean [16,60,61] and non-Mediterranean regions [43].

In addition, the daily habit of consuming ultra-processed plant-based food meat alternatives was identified as a further factor that enhances the likelihood of adopting a Mediterranean dietary pattern among Italian university students. Given the wider market availability of meat alternatives [62,63], the impact of these products on students’ diets has been more pronounced in the US [43]. However, their consumption per capita in Italy is expected to grow in the next years [62]. Though global recommendations suggest consuming plant-based foods in large quantities by favoring low-processed products [64], these meat alternatives might serve as a possible solution to nudge meat eaters to a more plant-based diet with less effort than eating whole plant foods, which often require higher preparation time and cooking skills. Moreover, as the intake of ultra-processed plant-based substitutes has not been associated with an increased risk of chronic metabolic diseases [65], it is advisable to prioritize the nutritional content of the product rather than its processing level [66]. When focusing on meat alternatives available in the European market, a certain variability in nutrient content can be found [63]. Overall, vegetarian and vegan alternatives in Europe contribute to fiber and protein intake and are generally lower in salt, saturated fat, and energy density than red meat products [67].

Government actions should prioritize the development of a supportive food environment that facilitates proper food choices and promotes population-wide healthy and sustainable eating. Recommended policies may include regulations to increase the nutritional quality of processed products, the establishment of food advertisement laws, the improvement and harmonization of different front-of-pack (FOP) nutrition labeling, and the implementation of specific criteria for food procurement in public institutions to guarantee that the food offered in public entities contributes to healthy and sustainable diets [68]. Concerning this last point, previous studies have emphasized the role of education in adopting sustainable dietary patterns [69] and the key role of universities in raising awareness among young adults about food sustainability and the environmental impact of food [70,71] through the creation of new interdisciplinary curricula and a healthy built environment, starting with the food services [50,59]. In line with this, in our study, students who attended the university canteen daily over the six months prior to the study were more adherent to the MD. However, the percentage of students attending university cafeterias daily in Italy was lower compared to the US [43], where a higher share of students live on campus. Efforts to make dining halls a supportive environment to foster good eating habits among the university community can be observed in the US, as many universities are involved in the Menus of Change University Research Collaborative (MCURC), a nationwide project aimed at promoting a healthy and sustainable food environment through campus food services [72]. From this perspective, future research could explore the effectiveness of different types of nudging techniques, for instance in terms of message contents and formats combining gain- or loss-framed information [73], as well as evaluate the results after a longer exposure.

Certainly, the gender and geographic distribution representativeness of the study population, as well as the administration of a validated instrument such as the KIDMED questionnaire, represent the main strengths of our work. In addition, the simultaneous use of the SHED index questionnaire allowed the expansion of the assessment of participants’ eating behaviors. However, to properly contextualize the results, some inherent limitations of the study design should be pointed out. To begin with, self-administered online surveys facilitate data collection and subject participation (given the little effort required) but increase the risks of recall bias and misreporting information. In this regard and being a cross-sectional study, this investigation aimed to provide a representative picture of the current eating habits of Italian university students, seeking to identify which potential changes might be the most relevant for improving their health.

Future intervention studies are needed to confirm the roles of the behavioral change determinants highlighted in this study. In addition, given the significant associations found between certain dietary behaviors and the university geographical location, future research should thoroughly explore potential regional differences within the country. This, together with information about ethnic background, would enable a better understanding of the influences of socio-cultural factors, would help with the identification of dietary behaviors that required greater attention based on the local context, and would support the development of effective initiatives to foster sustainable eating habits among young adults. In this context, our findings are not directly generalizable to other countries. Rather, our survey may serve as a starting point for developing future investigations in different settings, helping to identify country-specific characteristics.

Due to constraints related to the overall length of the questionnaire, some aspects were not fully investigated, such as food environment factors or the family history of dietary habits. A deeper exploration of these aspects would provide a more comprehensive understanding of the determinants of dietary behaviors, both in Italy and other countries.

Regarding the SHED index questionnaire, this tool has not yet been validated for the Italian population. The tool was developed in Israel [16] and recently validated in a sample of Portuguese adults [60], mostly young adults and women. Since both are populations from Mediterranean regions and considering the similarity between our sample and the one enrolled in Portugal, as well as the proportional positive association with the MD shown in all three studies, it can be assumed that it represents a reliable instrument even for our study population. Our analyses of the SHED index questionnaire’s reliability are consistent with the validation performed by the authors [16], showing higher internal consistency and stronger correlations with the total score for the HE, SE and BFV sub-scores. However, a specific validation study in the Italian context would be crucial to confirm the promising results of the present study and to evaluate any necessary adaptations of the tool. Besides validation in other populations, future developments of the questionnaire should refine the rating of the BFV sub-score by combining information on the fruit and vegetable purchase location with the concept of seasonality [74]. Like the SHED index questionnaire, the KIDMED questionnaire has some limitations that should be addressed, such as the combined use of fruit and fresh fruit juice in the first item, with the assumption of comparability between the two entries. Indeed, based on the evidence reported in the literature [75,76], the beneficial impact on health is widely ascertained for fruit [75] but not for juices [76]. Therefore, it would be advisable to rephrase the question excluding the wording “fresh fruit juice” or create an additional item to explore its consumption separately.

## 5. Conclusions

Italian university students showed an overall medium adherence to the MD. The most common aspects of the diet were the habit of having breakfast, eating fruit and vegetables at least once a day, consuming pasta or other cereals almost every day, and using olive oil at home. However, there is still plenty of scope for improvement in other aspects of the diet, such as for the second serving of fruit and vegetables, as well as for the intake of fish, nuts, legumes, and dairy products. Overall, students who had an active lifestyle, consumed a larger percentage of plant-based products, and had more sustainable dietary behaviors according to the SHED index score were also more likely to adopt healthier eating habits. Finally, it is worth remarking on the potential roles of innovative products such as ultra-processed plant-based meat alternatives and university food services in encouraging more sustainable diets among young generations. In this context, future food policy will be crucial in shaping a food environment that facilitates consumer choice and fosters sustainable food systems. In particular, government actions should aim at improving the quality of processed products, increasing the usability of label information, and ensuring proper food marketing. Lastly, given the poor attendance of university canteens in Italy, it is crucial to implement initiatives that encourage the university community to use campus dining services.

## Figures and Tables

**Table 1:** Participants’ characteristics reported for the total sample and by gender.

Variables	All (*n =* 1434)	Men (*n* = 562)	Women (*n* = 860)	Non-Binary Gender (*n* = 12)	*p*-Value
Age (years)	22.0 (20.0–23.0)	22.0 (20.0–23.0)	22.0 (20.0–23.0)	21.5 (20.3–24.0)	0.222 ^§^
Height (cm)	169.0 (162.0–175.0)	177.0 (171.0–180.0) ^a^	164.0 (160.0–169.0) ^b^	169.0 (158.5–176.5) ^b^	<0.001 ^§^ <0.001 ^§^ <0.001 ^§^ <0.001 ^†^
Body weight (kg)	64.0 (55.0–73.0)	72.0 (66.0–80.0) ^a^	58.0 (52.0–65.0) ^b^	61.5 (55.5–68.8) ^b^	
BMI (kg/m^2^)	22.2 (20.1–24.5)	23.0 (21.5–25.0) ^a^	21.3 (19.5–23.9) ^b^	22.3 (18.8–25.0) ^ab^	
BMI category					
Underweight	152 (10.6)	23 (4.1)	126 (14.7)	3 (25.0)	
Normal weight	984 (68.6)	400 (71.2)	577 (67.1)	7 (58.3)	
Overweight	243 (16.9)	119 (21.2)	123 (14.3)	1 (8.3)	
Obesity	55 (3.8)	20 (3.6)	34 (4.0)	1 (8.3)	
Geographical area of the university location					0.002 ^†^
Northeast	290 (20.2)	113 (20.1)	176 (20.5)	1 (8.3)	
Northwest	384 (26.8)	183 (32.6)	199 (23.1)	2 (16.7)	
Center	321 (22.4)	114 (20.3)	205 (23.8)	2 (16.7)	
South or Islands	439 (30.6)	152 (27.0)	280 (32.6)	7 (58.3)	
Geographical area of origin					<0.001 ^†^
Northwest	368 (25.7)	168 (29.9)	198 (23.0)	2 (16.7)	
Northeast	231 (16.1)	100 (17.8)	131 (15.2)	0 (0)	
Center	271 (18.9)	97 (17.3)	174 (20.2)	0 (0)	
South or Islands	564 (39.3)	197 (35.1)	357 (41.5)	10 (83.3)	
Educational stage					0.504 ^†^
Undergraduate student	924 (64.4)	368 (65.5)	546 (63.5)	10 (83.3)	
Graduate student	324 (22.6)	120 (21.4)	202 (23.5)	2 (16.7)	
Single cycle student	186 (13.0)	74 (13.2)	112 (13.0)	0 (0)	
Field of study					<0.001 ^†^
Food Science	234 (16.3)	150 (26.7)	84 (9.8)	0 (0)	
Medicine	135 (9.4)	36 (6.4)	96 (11.2)	3 (25.0)	
Scientific–Technological	384 (26.8)	170 (30.2)	212 (24.7)	2 (16.7)	
Human–Social	676 (47.1)	204 (36.3)	465 (54.1)	7 (58.3)	
Other	5 (0.3)	2 (0.4)	3 (0.3)	0 (0)	
Living place typology					<0.001 ^†^
On campus	78 (5.4)	48 (8.5)	29 (3.4)	1 (8.3)	
Off-campus by myself	77 (5.4)	43 (7.7)	34 (4.0)	34 (4.0)	
Off-campus with my partner	62 (4.3)	19 (3.4)	43 (5.0)	43 (5.0)	
Off-campus with my roommates	282 (19.7)	90 (16.0)	190 (22.1)	190 (22.1)	
Parents’ house	926 (64.6)	359 (63.9)	559 (65.0)	559 (65.0)	
Other	9 (0.6)	3 (0.5)	5 (0.3)	5 (0.3)	
Financial situation					0.003 ^†^
Never have to worry about money	258 (18.0)	123 (21.9)	153 (15.7)	0 (0)	
Worry about money for fun and extras	488 (34.0)	203 (36.1)	279 (32.4)	6 (50.0)	
Just enough to get by	421 (29.4)	151 (26.9)	267 (31.0)	3 (25.0)	
Not enough to get by	116 (8.1)	30 (5.3)	85 (9.9)	1 (8.3)	
I prefer not to answer	151 (10.5)	55 (9.8)	94 (10.9)	2 (16.7)	
Attendance at the university canteen in the last 6 months					<0.001 ^†^
Never/rarely	814 (56.8)	283 (50.4)	520 (60.5)	11 (91.7)	
<1 time/week	190 (13.2)	66 (11.7)	124 (14.4)	0 (0)	
1–2 times/week	216 (15.1)	92 (16.4)	123 (14.3)	1 (8.3)	
3–4 times/week	135 (9.4)	69 (12.3)	66 (7.7)	0 (0)	
5–6 times/week	54 (3.8)	36 (6.4)	18 (2.1)	0 (0)	
Once per day or more	25 (1.7)	16 (2.8)	9 (1.0)	0 (0)	
MVPA recommendations					<0.001 ^†^
Not met	728 (50.8)	224 (39.9)	494 (57.4)	10 (83.3)	
Met	706 (49.2)	338 (60.1)	366 (42.6)	2 (16.7)	
KIDMED score	6.0 (4.0–8.0)	6.0 (4.0–8.0)	6.0 (4.0–8.0)	6.0 (4.0–7.8)	0.077 ^§^
Level of adherence to the MD ^a^					0.146 ^†^
Low	246 (17.2)	86 (15.3)	160 (18.6)	0 (0)	
Medium	789 (55.0)	307 (54.6)	473 (55.0)	9 (75.0)	
High	399 (27.8)	169 (30.1)	227 (26.4)	3 (25.0)	
SHED index score	68.0 (55.0–81.0)	65.0 (51.0–79.3) ^b^	69.0 (56.0–82.0) ^a^	65.5 (52.0–84.5) ^ab^	0.005 ^§^
SHED sub-scores					
HE score	17.0 (13.0–20.0)	15.0 (12.0–19.0) ^b^	18.0 (14.0–21.0) ^a^	20.0 (17.3–20.8) ^a^	<0.001 ^§^
SE score	13.0 (11.0–15.0)	13.0 (11.0–14.0) ^b^	18.0 (14.0–21.0) ^a^	16.0 (11.3–18.3) ^ab^	<0.001 ^§^
BFV score	28.0 (20.0–38.0)	29.0 (20.0–40.0)	28.0 (20.0–37.0)	29.0 (22.0–39.5)	0.426
Ready meals score	16.0 (12.0–20.0)	14.0 (11.0–19.0) ^b^	17.0 (13.3–21.0) ^a^	15.0 (11.0–18.8) ^ab^	<0.001 ^§^
Water score	2.0 (−3.0–6.0)	2.0 (−3.0–6.0)	2.0 (−3.0–7.0)	−2.0 (−4.5–5.8)	0.509 ^§^
Soda score	−9.0 (−12.0–−7.0)	−9.0 (−11.0–−6.0) ^a^	−10.0 (−13.0–−8.0) ^b^	−11.5 (−12.0–7.8) ^ab^	<0.001 ^§^
Self-reported dietary pattern					<0.001 ^†^
Omnivore	1285 (89.6)	538 (95.7)	739 (85.9)	8 (66.7)	
Plant-based ^b^	143 (10.0)	23 (4.1)	116 (13.5)	4 (33.3)	
Others ^c^	6 (0.4)	1 (0.2)	5 (0.6)	0 (0)	
% Plant-based foods in the diet	50.0 (38.0–67.3)	47.5 (35.0–60.3) ^b^	53.0 (40.0–70.0) ^a^	64.0 (43.8–92.3) ^ab^	<0.001 ^§^
Willingness to purchase and consume healthy and sustainable dishes					<0.001 ^†^
Yes	1015 (70.8)	349 (62.1)	655 (76.2)	11 (91.7)	
No	419 (29.2)	213 (37.9)	205 (23.8)	1 (8.3)	
Frequency of eating ultra-processed plant-based meat alternative foods					<0.023 ^†^
Never/Rarely	652 (45.5)	275 (48.9)	370 (43.0)	7 (58.3)	
1–2 times/month	310 (21.6)	108 (19.2)	200 (23.3)	2 (16.7)	
≤1 time/week	279 (19.5)	101 (18.0)	178 (20.7)	0 (0)	
2–3 times/week	146 (10.2)	62 (11.0)	82 (9.5)	2 (16.7)	
4–5 times/week	33 (2.3)	14 (2.5)	19 (2.2)	0 (0)	
Daily or almost daily	14 (1.0)	2 (0.4)	11 (1.3)	1 (8.3)	

The data are presented as the medians (IQRs) for continuous variables and as numbers (%) for categorical variables. ^§^ Nonparametric Kruskal–Wallis H test for independent sample pairwise comparison. Different letters in the same line denote significant differences. ^†^ Pearson’s Chi-square test. BMI: Body Mass Index; MD: Mediterranean diet; MVPA: Moderate to Vigorous Physical Activity; SHED: Sustainable Healthy Diet; HE: Healthy Eating; SE: Sustainable Eating; BFV: Fruits and Vegetable Purchasing Location. ^a^ Low total score ≤ 3 points; medium total score of 4–7 points; high total score ≥ 8 points. ^b^ Including vegetarian, vegan, flexitarian, pescetarian, and fruitarian dietary patterns. ^c^ Including raw foodism and unspecified dietary patterns. Single-cycle degree program—“Laurea Magistrale a ciclo unico”. These programs provide a master’s degree after a single cycle of 5 or 6 years in various disciplines regulated by special European protocols (e.g., medicine, veterinary medicine, law, and architecture).

**Table 2:** Socio-demographic characteristics, anthropometric data, and food-related habits reported by level of adherence to the MD.

Variables	Adherence to the MD ^a^			
	Low (*n* = 246)	Medium (*n* = 789)	High (*n* = 399)	*p*-Value
Age (years)	21.0 (20.0–23.0) ^b^	22.0 (21.0–23.0) ^a^	22.0 (21.0–23.0) ^a^	0.001 ^§^
BMI (kg/m^2^)	21.9 (19.6–24.2)	22.3 (20.1–24.9)	22.2 (20.2–24.2)	0.090 ^§^ 0.031 ^†^
BMI category				
Underweight	30 (12.2)	77 (9.8)	45 (11.3)	
Normal weight	172 (69.9)	523 (66.3)	289 (72.4)	
Overweight	33 (13.4)	153 (19.4)	57 (14.3)	
Obesity	11 (4.5)	36 (4.6)	8 (2.0)	
Geographical area of the university location				0.145 ^†^
Northeast	40 (16.3)	162 (20.5)	88 (22.1)	
Northwest	60 (24.4)	204 (25.9)	120 (30.1)	
Center	58 (23.6)	179 (22.7)	84 (21.1)	
South or Islands	88 (35.8)	244 (30.9)	107 (26.8)	
Geographical area of origin				0.058 ^†^
Northeast	59 (24.0)	202 (25.6)	107 (26.8)	
Northwest	26 (10.6)	129 (16.3)	76 (19.0)	
Center	47 (19.1)	156 (19.8)	68 (17.0)	
South or Islands	114 (46.3)	302 (38.3)	148 (37.1)	
Educational stage				0.002 ^†^
Undergraduate student	184 (74.8)	501 (63.5)	239 (59.9)	
Graduate student	35 (14.2)	186 (23.6)	103 (25.8)	
Single-cycle student	27 (11.0)	102 (12.9)	57 (14.3)	
Field of study				0.135 ^†^
Food	32 (13.0)	124 (15.7)	78 (19.5)	
Medicine	16 (6.5)	84 (10.6)	35 (8.8)	
Scientific–Technological	65 (26.4)	214 (27.1)	105 (26.3)	
Human–Social	131 (53.3)	365 (46.3)	26.6 (45.1)	
Other	2 (0.8)	2 (0.3)	1 (0.3)	
Living place typology				0.321 ^†^
On campus	6 (2.4)	48 (6.1)	24 (6.0)	
Off-campus by myself	9 (3.7)	46 (5.8)	22 (5.5)	
Off-campus with my partner	12 (4.9)	30 (3.8)	20 (5.0)	
Outside campus with roommates	56 (22.8)	147 (18.6)	79 (19.8)	
Parents’ house	163 (66.3)	512 (64.9)	251 (62.9)	
Other	0 (0.0)	6 (0.8)	3 (0.8)	
Financial situation				0.022 ^†^
Never have to worry about money	25 (10.2)	151 (19.1)	139 (34.8)	
Worry about money for fun and extras	81 (32.9)	268 (34.0)	105 (26.3)	
Just enough to get by	87 (35.4)	229 (29.0)	30 (7.5)	
Not enough to get by	20 (8.1)	66 (8.4)	43 (10.8)	
I prefer not to answer	33 (13.4)	75 (9.5)	82 (20.6)	
Attendance at the university canteen in the last 6 months				0.059 ^†^
Never/rarely	152 (61.8)	450 (57.0)	212 (53.1)	
<1 time/week	37 (15.0)	97 (12.3)	56 (14.0)	
1–2 times/week	35 (14.2)	118 (15.0)	63 (15.8)	
3–4 times/week	18 (7.3)	80 (10.1)	37 (9.3)	
5–6 times/week	2 (0.8)	33 (4.2)	19 (4.8)	
Once per day or more	2 (0.8)	11 (1.4)	12 (3.0)	
MVPA recommendations				<0.001 ^†^
Not met	156 (63.4)	427 (54.1)	145 (36.3)	
Met	90 (36.6)	362 (45.9)	254 (63.7)	
Dietary pattern				<0.001 ^†^
Omnivore	236 (95.9)	717 (90.9)	332 (83.2)	
Plant-based ^b^	8 (3.3)	70 (8.9)	65 (16.3)	
Others ^c^	2 (0.8)	2 (0.3)	2 (0.5)	
% Plant-based foods in the diet	35.0 (20.8–45.0) ^c^	50.0 (40.0–66.0) ^b^	60.0 (49.0–73.0) ^a^	<0.001 ^§^
Willingness to purchase and consume healthy and sustainable dishes				<0.001 ^†^
Yes	121 (49.2)	560 (71.0)	334 (83.7)	
No	125 (50.8)	229 (29.0)	65 (16.3)	
Frequency of eating plant-based ultra-processed meat alternative foods				0.003 ^†^
Never/Rarely	136 (55.3)	355 (45.0)	161 (40.4)	
1–2 times/month	54 (22.0)	171 (21.7)	85 (21.3)	
≤1 time/week	35 (14.2)	154 (19.5)	90 (22.6)	
2–3 times/week	17 (6.9)	86 (10.9)	43 (10.8)	
4–5 times/week	3 (1.2)	19 (2.4)	11 (2.8)	
Daily or almost daily	1 (0.4)	4 (0.5)	9 (2.3)	

The data are presented as the medians (IQRs) for continuous variables and as numbers (%) for categorical variables. ^§^ Nonparametric Kruskal–Wallis H test for independent sample with pairwise comparisons. Different letters in the same line denote significant differences among adherence to MD groups. ^†^ Pearson’s Chi-square test. BMI: Body Mass Index; MVPA: Moderate to Vigorous Physical Activity. ^a^ Low total score ≤ 3 points; medium total score of 4–7 points; high total score ≥ 8 points. ^b^ Including vegetarian, vegan, flexitarian, pescetarian, and fruitarian dietary patterns. ^c^ Including raw foodism and unspecified dietary patterns.

**Table 3:** Logistic regression analyses for having high adherence to the MD (KIDMED score ≥ 8 points).

	Univariate Analysis		Multivariate Analysis	
Variables	OR (95% CI)	*p*-Value	OR (95% CI)	*p*-Value
Age	1.069 (0.992–1.152)	0.078	1.035 (0.937–1.143)	0.499
BMI (kg/m^2^)				
18.5–24.9 (normal weight)	−1−		−1−	
<18.5 (underweight)	1.011 (0.696–1.470)	0.953	1.186 (0.773–1.820)	0.435
25.0–29.9 (overweight)	0.737 (0.531–1.022)	0.067	0.729 (0.493–1.076)	0.111
30.0–34.9 (obesity)	0.409 (0.191–0.877)	0.022	0.371 (0.151–0.909)	0.030
Educational stage				
Undergraduate student	−1−		−1−	
Graduate student	1.336 (1.013–1.761)	0.040	1.108 (0.768–1.598)	0.584
Other (single-cycle degree)	1.266 (0.897–1.788)	0.179	1.127 (0.734–1.731)	0.585
Financial situation				
Not enough to get by	−1−		−1−	
Just enough to get by	0.953 (0.595–1.525)	0.839	0.973 (0.577–1.640)	0.917
Never have to worry about money	1.142 (0.721–1.808)	0.572	1.206 (0.726–2.003)	0.468
Worry about money for fun and extras	1.336 (0.817–2.183)	0.248	1.167 (0.672–2.029)	0.583
Attendance at the university canteen in the last 6 months				
Never/rarely	−1−		−1−	
<1 time/week	1.187 (0.837–1.682)	0.336	1.233 (0.824–1.845)	0.308
1–2 times/week	1.169 (0.838–1.631)	0.357	1.088 (0.738–1.603)	0.672
3–4 times/week	1.072 (0.712–1.614)	0.739	0.860 (0.520–1.421)	0.556
5–6 times/week	1.542 (0.863–2.753)	0.144	0.763 (0.390–1.495)	0.431
Once per day or more	2.621 (1.178–5.834)	0.018	2.311 (0.893–5.978)	0.084
MVPA recommendations				
Not met	−1−		−1−	
Met	2.259 (1.781–2.867)	<0.001	2.377 (1.800–3.141)	<0.001
SHED index score	1.028 (1.022–1.035)	<0.001	1.017 (1.009–1.026)	<0.001
Dietary pattern				
Omnivore	−1−		−1−	
Plant-based ^a^	2.392 (1.682–3.402)	<0.001	1.389 (0.890–2.169)	0.148
Others ^b^	1.435 (0.262–7.872)	0.677	1.054 (0.121–9.156)	0.962
% Plant-based foods in the diet	1.028 (1.022–1.034)	<0.001	1.021 (1.013–1.029)	<0.001
Willingness to purchase and consume healthy and sustainable dishes				
No/maybe	−1−		−1−	
Yes	2.671 (1.988–3.588)	<0.001	1.639 (1.153–2.329)	0.006
Frequency of eating plant-based ultra-processed meat alternative foods				
Never/Rarely	−1−		−1−	
1–2 times/month	1.152 (0.848–1.565)	0.365	0.843 (0.588–1.208)	0.352
≤1 time/week	1.452 (1.068–1.976)	0.017	0.798 (0.549–1.160)	0.237
2–3 times/week	1.273 (0.855–1.895)	0.234	0.613 (0.378–0.994)	0.047
4–5 times/week	1.525 (0.724–3.213)	0.267	0.851 (0.360–2.016)	0.714
Daily or almost daily	5.489 (1.813–16.617)	0.003	3.209 (0.742–13.888)	0.119

BMI: Body Mass Index; SHED: Sustainable Healthy Diet; MVPA: Moderate to Vigorous Physical Activity. ^a^ Including vegetarian, vegan, flexitarian, pescetarian, and fruitarian dietary patterns. ^b^ Including raw foodism and unspecified dietary patterns.

## Data Availability

The raw data supporting the conclusions of this article will be made available by the authors upon request.

## Supplementary Materials

The following supporting information can be downloaded at https://www.mdpi.com/article/10.3390/nu17121988/s1, Table S1. STROBE-nut checklist; Table S2. SHED sub-scores’ correlations with the total score and internal consistency; Figure S1. Subjects’ distribution for each item of the KIDMED questionnaire reported by gender; Figure S2. Subjects’ distribution for each item of the KIDMED questionnaire reported by geographical area of university location; and Figure S3. Subjects’ distribution based on the answer option selected for each item of the SHED index questionnaire reported by gender.

## Abbreviations

The following abbreviations are used in this manuscript:

MD	Mediterranean diet
PHD	Planetary Health Diet
SHED	Sustainable Healthy Diet
HE	Healthy Eating
SE	Sustainable Eating
BFV	Fruits and Vegetable Purchasing Location
NPAQ	Nordic Physical Activity Questionnaire
MVPA	Moderate to Vigorous Physical Activity
VPA	Vigorous Physical Activity
FOP	Front of Pack
